# Quantitative genetics of learning ability and resistance to stress in *Drosophila melanogaster*

**DOI:** 10.1002/ece3.1379

**Published:** 2015-01-07

**Authors:** Virginie Nepoux, Aurélie Babin, Christoph Haag, Tadeusz J Kawecki, Arnaud Le Rouzic

**Affiliations:** 1 Department of Ecology and Evolution, University of LausanneLausanne, CH-1015, Switzerland; 2 Centre d'Écologie Fonctionnelle et Évolutive, UMR 5175, CNRS – Université de Montpellier – Université Paul-Valéry Montpellier – EPHAMontpellier 5, FR-34293, France; 3 Laboratoire Evolution Génome et Spéciation, UPR 9034, CNRSGif-sur-Yvette, FR-91198, France

**Keywords:** Additive genetic variation, diallel crossing, *Drosophila melanogaster*, genetic correlation, learning

## Abstract

Even though laboratory evolution experiments have demonstrated genetic variation for learning ability, we know little about the underlying genetic architecture and genetic relationships with other ecologically relevant traits. With a full diallel cross among twelve inbred lines of *Drosophila melanogaster* originating from a natural population (0.75 < *F* < 0.93), we investigated the genetic architecture of olfactory learning ability and compared it to that for another behavioral trait (unconditional preference for odors), as well as three traits quantifying the ability to deal with environmental challenges: egg-to-adult survival and developmental rate on a low-quality food, and resistance to a bacterial pathogen. Substantial additive genetic variation was detected for each trait, highlighting their potential to evolve. Genetic effects contributed more than nongenetic parental effects to variation in traits measured at the adult stage: learning, odorant perception, and resistance to infection. In contrast, the two traits quantifying larval tolerance to low-quality food were more strongly affected by parental effects. We found no evidence for genetic correlations between traits, suggesting that these traits could evolve at least to some degree independently of one another. Finally, inbreeding adversely affected all traits.

## Introduction

Learning, that is the ability to modify behavior based on experience (Rescorla 1988; Papaj and Prokopy 1989), is thought to be generally adaptive, in particular in variable environments (Shettleworth 1998; Dukas 1998; Kawecki 2010; Danchin et al. 2010). Still, most animals exhibit only moderate learning abilities. Three hypotheses can be proposed to explain the evolutionary stasis of a phenotypic character: (1) the lack of directional selection pressure, (2) the lack of additive genetic variation for this character, and (3) physiological or ecological trade-offs generating fitness costs that are higher than the benefits of evolving the trait. The two last explanations rely on the genetic architecture underlying phenotypic variation in learning ability. The maintenance of genetic variation for fitness-related traits can be facilitated by dominance and epistatic interactions between polymorphic loci (Gimelfarb 1989). In this scenario, genetic variation exists, but its additive contribution is small. Epistasis has been found to contribute significantly to life-history traits in many studies (reviewed by Roff and Emerson 2006). However, little is known about the contribution of epistasis to genetic variation for learning performance, although a pattern of crosses between replicate lines selected for improved learning performance suggested a strong epistatic component (Kawecki and Mery 2006). Similarly, little is known about the contribution of maternal effects to the genetic architecture of learning ability, even though there is evidence for the effect of maternal age on offspring learning performance (Burns and Mery 2010).

Alternatively, evolution of learning may be limited by indirect negative selection due to antagonistic genetic correlations with fitness components. Cognitive processes are energetically costly, in particular under nutritional limitation or other forms of physiological stress. Such physiological trade-offs in turn may (although need not) lead to evolutionary, genetically based trade-offs (Stearns 1989). Some selection experiments in *Drosophila* detected apparent evolutionary trade-offs between learning performance and other fitness-related traits, such as longevity (Burger et al. 2008) and the ability to compete for highly limited food (Mery and Kawecki 2003). In parallel, a physiological link between learning and tolerance to nutritional stress has been suggested. Jaumann et al. (2013) showed that starved bees displayed poorer learning, and long-term memory formation is known to reduce tolerance to starvation in *Drosophila* (Mery and Kawecki 2005). Adverse effects of infection or immune system activation on learning performance in rodents (Kavaliers et al. 1995; Gibertini et al. 1995; Sparkman et al. 2005) and bees (Mallon et al. 2003; Gegear et al. 2006) also point to similar physiological links between learning and immune defense (although see Babin et al. 2014b or an opposite result).

In this study, we investigated these aspects of the genetic architecture of aversive olfactory learning performance in flies derived from a natural population of *D. melanogaster*, in conjunction with fitness-related traits previously implicated in trade-offs with learning: tolerance to malnutrition (Mery and Kawecki 2003, 2005; Nepoux et al. 2010) and immune defense (Mallon et al. 2003; Gegear et al. 2006). We employed a complete diallel cross-design (Griffing 1956), crossing each of 12 inbred lines with all others, including with itself. With this experiment, we aim to address three specific questions: (1) What is the genetic architecture of these traits in terms of variance components attributable to additive genetic, cross-specific, and parental contributions? Strong dominance or epistatic effects could suggest that the genetic variation may be partly maintained by balancing selection. (2) Is there evidence for genetic correlations between learning and life-history traits? Negative correlations would support the existence of genetic trade-offs, explaining why better learning cannot evolve in natural populations. (3) To what extent are these traits affected by inbreeding? Inbreeding depression is associated with nonadditive genetic architectures, and strong inbreeding effects could generate different genetic correlations when measured from inbred or outbred animals.

## Material and Methods

### Inbred lines and cross-design

The lines originated from a population of 400 flies collected in Valais (Switzerland) in October 2007. They were generated by transferring a single mated female in a fresh vial over 12 generations. At the end of the process, the inbreeding coefficient was at least 0.75 (half-sib matings) and at most 0.93 (full-sib matings) (Nepoux et al. 2010). They had subsequently been maintained at 200 to 300 individuals per line on a standard food medium with 8% yeast (David and Clavel 1965) and under standard laboratory conditions (25°C, 60% relative humidity, 12:12 light:dark cycle). Fifty lines have been established in the beginning, but most have died out during or after the inbreeding phase. The twelve remaining lines have been tested in this study.

To obtain the 144 crosses of the full diallel matrix (12 × 12 lines), each of the twelve inbred lines was crossed with all the others (132 outbred crosses) and with itself (12 inbred crosses). All the crosses between different lines were thus performed in both directions (reciprocal crosses). For each cross, eggs were collected from 15 one-week-old virgin females of the mother line mated with 10 one-week-old males of the father line. For logistic reasons, the diallel table was split into two blocks of 72 crosses each, set up and tested on two different days. All crosses were replicated twice (each replicate measurement was based on many flies, as described below).

### Phenotypic assays

Tolerance to malnutrition was measured as the developmental rate and egg-to-adult viability of larvae raised on a low-quality food with diluted nutritional content; immune defense was quantified as survival of a systemic infection with the pathogenic bacterium *Pseudomonas entomophila*; short-term memory was measured as an estimation of learning performance. Because learning performance can be affected by the sensory perception of the stimuli involved, we also analyzed unconditioned responses to odors. Individuals tested for learning performance, viability, and developmental rate were produced from the same generation of parents, while resistance to infection and the unconditioned response to odors were measured on individuals produced from the next generation of parents.

#### Learning performance

Groups of 5- to 7-day-old flies (mixed sexes) were tested for learning performance in an aversive olfactory conditioning based on the avoidance of one odorant previously associated with an aversive mechanical shock (Mery and Kawecki 2005; Mery et al. 2007). After emergence, flies were split into two subgroups of similar sizes (approximately 50 individuals, less than 50 for the crosses that did not produce enough eggs) under CO_2_ anesthesia and let recover for 24 h. Flies were then transferred without anesthesia to test tubes. The conditioning procedure consisted of three back-to-back conditioning cycles. In each cycle, the flies were first exposed to one odorant for 30 s, coupled with 1 s pulses of mechanical shock every 4 s. This was followed by 60 s of humid air; a second odorant was then delivered for 30 s without shock, followed by another 60 s of humid air. The odorants were 3-octanol (OCT, 0.6 mL/L) and 4-methylcyclohexanol (MCH, 0.6 mL/L) dissolved in paraffin oil. Memory retrieval was tested by allowing flies to choose for 60 s between the two odors in a T-maze. Memory was tested 2 to 6 min after the end of conditioning, which corresponds to short-term memory (Margulies et al. 2005). One subset of flies was conditioned to avoid MCH, while the other subset was conditioned to avoid OCT. Flies in each arm of the T-maze were counted; flies which remained in the center of the maze were excluded.

#### Unconditioned response to odorants

We measured the response of flies to the odorants MCH and OCT (same concentrations as mentioned above) in the absence of conditioning. In the absolute preference test, naive flies were offered the choice between one odorant (either MCH or OCT) and solvent (paraffin oil). In the relative preference test, naive flies were offered the choice between the two odorants. 5 min prior to the preference tests, the flies were subject to the same amount of mechanical shock as during a 3-cycle conditioning to control for an effect of mechanical shock on odorant perception. Proportions of flies which chose the odorant in the absolute preference test, and which chose OCT in the relative preference test, were used as preference measures.

#### Resistance to bacterial infection

Groups of 30 mated females were collected under CO_2_ anesthesia and let to recover for 24 h on regular food. Systemic bacterial infection was performed under CO_2_ anesthesia by pricking flies on the thorax side with a thin needle (ø 0.15 mm) coated with a bacterial suspension (1/4 of 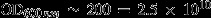 cells per mL suspended in 0.9% saline buffer) of the highly virulent generalist entomopathogen *Pseudomonas entomophila*, a natural Gram-negative bacterial pathogen of fruit flies (Vodovar et al. 2005). *P. entomophila* is one of the few bacterial pathogens which were reported as able to infect flies and elicit an immune response via the oral route. In this study, fruit flies were infected systemically with a strain isolated from Drosophila caught in the field on the French Caribbean island Guadeloupe about a decade ago (Vodovar et al. 2005) through pricking, which also elicits an immune response in the hemocoel (Babin et al. 2014a). Upon systemic infection with *P. entomophila*, the core immune response is mediated by the induction of the *imd* signaling pathway for the production of antimicrobial peptides, which is specific to Gram-negative bacteria (Lemaitre and Hoffmann 2007). While using a Gram-positive bacterial pathogen would induce another signaling pathway (*Toll*), using another Gram-negative bacterial pathogen would not change the core response, except maybe in its amplitude.

Survival was then measured every 8 h for 4 days. Proportion of flies alive at the last time point of the experiment was used as measure of resistance to infection. As a control for pricking-induced mortality, an additional group of about 10 females per cross was pricked with 0.9% saline buffer. This treatment allows to control for the effect of pricking itself, that is piercing a hole in the fly's cuticle that itself elicits a wound healing response by the immune system. Mortality after sham pricking was 3% in average after 172 h (5% in inbred crosses, the difference not being statistically significant). Mortality was evenly distributed among line pairs and was not specific from a dam/sire line (no genetic basis). Mortality after sham pricking was about an order of magnitude lower than the mortality observed after bacterial inoculation. Most of the mortality of the sham pricked flies is likely to reflect infection with ambient bacteria present on the cuticle, so we did not normalize the mortality of *P. entomophila*-pricked flies by the mortality of sham controls of the same cross.

#### Tolerance to larval malnutrition

Tolerance to malnutrition was assayed as egg-to-adult viability and developmental rate of larvae developing on a food medium containing only 0.8% of yeast w/v (1/10 of the concentration of the medium used for line maintenance). Following the approach described in Nepoux et al. (2010), groups of 100 eggs were transferred to 60-mL vials on 10 mL of food; infertile (transparent) and damaged eggs were excluded from the collection. Some crosses did not provide enough eggs, and eggs were collected by several different experimenters; these factors were taken into account in data analysis. Newly emerged adults were counted every day for 14 days. For each vial, we calculated the mean developmental rate and an estimate of egg-to-adult viability (i.e., the proportion of eggs that resulted in emerged adults); these values were used as data in the analysis.

### Data analysis

#### Variance components estimation

The analysis of the progeny of crosses between inbred lines derived from a natural population allows estimating the genetic variance components of this population. Sprague and Tatum (1942) defined two sources of genetic variation: (1) the general combining ability of each line (GCA), which corresponds to half its breeding value (Wricke and Weber 1986; Falconer and Mackay 1996) and (2) the specific combining ability for each cross (SCA), defined as the deviation between the observed phenotypic value of the progeny and the phenotypic value expected from the breeding values of the parental lines. In addition, the differences between reciprocal crosses can be used to estimate general parental effects (RGCA), including cytoplasmic, epigenetic, and imprinting effects, and specific reciprocal effects (RSCA), featuring nuclear-by-cytoplasmic interactions.

Our analysis is based on a maximum-likelihood version of the Bayesian framework described in Lenarcic et al. (2012). This model allows to separate the GCA from parental effects. It uses a different parameterization than earlier models (Griffing 1956; Cockerham and Weir 1977; Greenberg et al. 2010), improving its statistical properties while remaining interpretable biologically. The resulting model features both fixed and random effects.

Keeping a similar notation as in Lenarcic et al. (2012), the expected phenotype of the cross between a female from line *i* and a male from line *j* is:

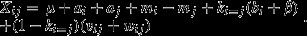


The full mixed-effect genetic model (corresponding to the “Babmvw" model in Lenarcic et al. 2012) is thus defined by two fixed effects (the intercept *μ* and the inbreeding effect *β*) and five random effect variances (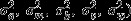. In this model, 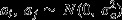 are the additive genetic contributions of lines *i* and *j*, 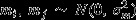 are the general parental effects (i.e., nongenetic parental effects averaged across crosses involving line *i*), 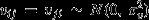 are the gene-specific effects (genetic interactions between lines *i* and *j*), and 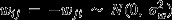 are the specific reciprocal effects. The variance of reciprocal effects 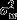 corresponds to the residual reciprocal variance, once the main parental effect has been removed. Inbreeding (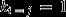 if *i* = *j*, 0 otherwise) is modeled by (1) a fixed change in the phenotype, *β*, corresponding to the average between outbred and inbred crosses and (2) a strain-specific random effect 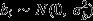. In theory, both parents can affect offspring phenotype through epigenetic effects on gene expression, as DNA methylation seems to play a role in *Drosophila* (Zemach et al. 2010).

Assuming complete homozygosity of parental lines and neglecting epistasis (which cannot be estimated without the phenotypic values of 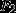 progenies), GCA and SCA variances (respectively, 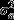 and 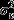) correspond to (Falconer and Mackay 1996; Lynch and Walsh 1998):

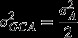


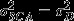


In reality, additive-by-additive epistasis and cytoplasmic or maternal inheritance affect the mean genetic value of parental line, therefore generating GCA (Falconer and Mackay 1996; Lynch and Walsh 1998). Similarly, epistasis contributes to SCA variance, in addition to dominance. As a consequence, diallel models (including ours) cannot estimate the additive genetic variance directly (i.e., 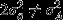), although these quantities are not independent.

**Figure 1:**
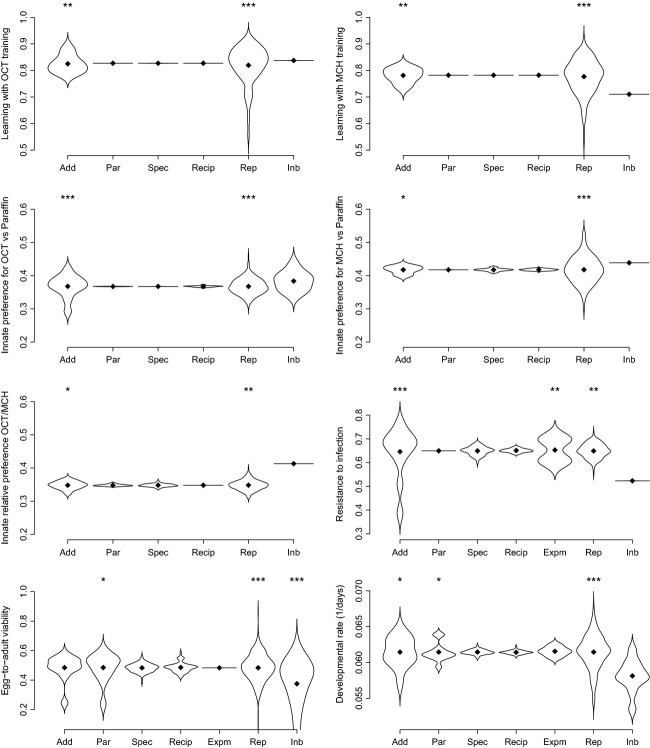
Estimates for additive genetic effects (Add), parental effects (Par), genetic specific effects (Spec), reciprocal effects (Recip), experimenter effects, when relevant (Expm), replicate effects (Rep), and inbreeding effects (Inb). Random effect predictors were back-transformed to the original scale, so that the y axis can be interpreted as probabilities, except for developmental rate (1/days). Distributions of random effects are centered (black diamonds) around their expectation (*μ* + *β* for inbreeding effect, *μ* for all other effects). The distributions of random effects are illustrated by violin plots, estimated with the “density" function in R, with default parameters (Sheather and Jones 1991; R Core Team 2014). The stars reflect the impact of removing individual effects (as in backward model selection) in terms of cAIC differences: *** ΔAIC > 10; ** ΔAIC > 5; * ΔAIC > 2.

Learning, innate preference, survival to infection, and egg-to-adult viability on poor food were considered as binomial traits and analyzed in a generalized linear model (GLM) framework, while development rate was treated as a Gaussian character. In addition to those genetic factors, additional parameters were included in the model for some traits: experimenter and replicate effects (Fig. 1).

For the learning ability, for a fly from maternal line *i*, from paternal line *j*, conditioned to avoid odorant *c* (*c* = 0 for MCH and 1 for OCT) in replicate *r*, the learning probability, a binomial trait (*y* = {0,1} whether or not the fly made the right choice), was modeled as:

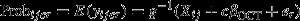


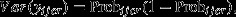
where 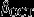, considered as a fixed effect, represents the effect of training for preference toward OCT (vs. MCH), and 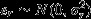 is the replicate effect, corresponding to the expected difference between two learning experiments performed in the same conditions. The link function *g* is the Gaussian cumulative distribution function (probit model). The statistical setting used to analyze the learning probability sensibly differs from the literature (Tully et al. 1994; Dubnau and Tully 1998; Mery and Kawecki 2005). Commonly, learning is reported as an index 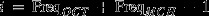, where Freq is the probability to make the good choice depending on the molecule associated with the mechanical shock. Here, we have modeled the learning frequency as 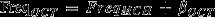, introducing a fixed effect 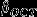 to account for the difference in learning ability depending on the molecule associated with the shock. This last setting allows a direct integration into a binomial GLM framework and therefore is a better model for the observation of variance. The three innate preference traits (OCT vs. Paraffin, MCH vs. Paraffin, and OCT vs. MCH) were modeled in the same way, without the 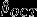 fixed effect.

Egg-to-adult viability was analyzed with a very similar model (*y* = {0,1} for development failure or success), but an “experimenter" effect was added to account for potential biases in the ability to determine the fertilization status of the eggs and potential damage during egg transfer. The full model thus becomes:

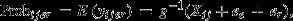
with 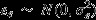 representing the effect of experimenter *e*. The same model was used to analyze survival data (*y* = {0, 1}). Finally, developmental time was considered to match a linear model with Gaussian residuals, implemented as:

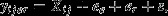
*y* being the observed developmental rate (in 1/days), and *ɛ* representing the residual error within each replicate.

These generalized linear mixed-effect models were fitted with extended quasi-likelihood method (quasi-REML), calculated from the “hglm" package version 1.8 (Rönnegård et al. 2010) for the R software version 3.0.1 (R Core Team 2014).

For each trait, we obtained the effects and their corresponding variance components on the transformed scale (probit scale for binomial traits).

It is now acknowledged that the Akaike information criterion is not well suited for model selection among mixed-effect models (Burnham and Anderson 2002; Vaida and Blanchard 2005). Here, we used the conditional AIC (cAIC), designed to handle such cases, which is available in the “hglm" package (Rönnegård et al. 2010).

#### Additive genetic correlations between traits

Additive genetic correlations were calculated for each pair of traits by extracting the random effect estimates (best linear unbiased predictors – BLUPs – and their nonlinear equivalents for the GLMs) of additive genetic effects estimated from the previous models. Significance of the correlations was tested with a Pearson's correlation test. Genetic effects used for the correlations were calculated on the transformed (probit) scale for the binomial traits.

## Results

### Variance partitioning

#### Learning ability and innate odorant preferences

Learning was found to vary significantly among lines; the probability of making the good odor choice after training ranged from 73 to 83%. The additive genetic contribution was the major source of variation for learning ability, for both conditioning directions (Fig. 1, Table 1). There was no contribution of genetic specific effects and reciprocal effects, or of parental effects. A separate analysis of data of each conditioning direction yielded similar values as the joint analysis of both directions (Table1). All traits but developmental rate being probabilities, their residual variance is fixed (and not estimated by the model) and cannot be compared meaningfully across traits. However, variances can be fairly compared to the replicate variance, corresponding to the unexplained variation between identical experiments.

**Table 1:** Partitioning of the variance of random effects (estimates with approximate 95% confidence intervals assuming a log-normal error on variances) into an additive genetic effect of parental lines (half breeding values, 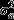), an inbreeding effect 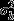, a parental effect (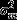), a genetic specific effect (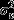), a reciprocal effect (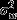), an experimenter effect (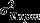) when measured, and replicate variance (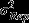). For all traits except developmental rate, the estimates of variances are given as (squared) probit-transformed probabilities. Developmental rate variances are given in 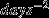

	a	b	m	v	w	Expm	Rep
Learning	0.011 (0.004, 0.028)	0.000	0.000	0.000	0.000		0.113 (0.097, 0.132)
Learn. OCT	0.023 (0.009, 0.056)	0.000	0.000	0.000	0.000		0.117 (0.094, 0.147)
Learn. MCH	0.011 (0.004, 0.029)	0.000	0.000	0.000	0.000		0.087 (0.069, 0.109)
Inn. OCT	0.009 (0.004, 0.023)	0.016 (0.004, 0.072)	0.000	0.000	0.001 (0.000, 0.004)		0.015 (0.010, 0.021)
Inn. MCH	0.002 (0.001, 0.005)	0.000	0.000	0.002 (0.000, 0.006)	0.001 (0.000, 0.005)		0.026 (0.020, 0.035)
Inn. pref.	0.002 (0.001, 0.005)	0.000	0.000	0.002 (0.001, 0.005)	0.000		0.009 (0.006, 0.014)
Survival	0.082 (0.035, 0.195)	0.000	0.000	0.014 (0.006, 0.032)	0.006 (0.002, 0.018)	0.029 (0.002, 0.415)	0.039 (0.026, 0.059)
Viability	0.057 (0.024, 0.136)	0.673 (0.277, 1.633)	0.070 (0.030, 0.165)	0.017 (0.009, 0.031)	0.020 (0.011, 0.036)	0.000	0.099 (0.080, 0.122)
Development	0.336 (0.142, 0.797)	1.287 (0.460, 3.598)	0.131 (0.054, 0.322)	0.049 (0.022, 0.110)	0.051 (0.023, 0.112)	0.054 (0.011, 0.275)	0.650 (0.536, 0.789)

The variation in innate preference ranged from 40 to 43% for MCH versus paraffin, from 29 to 42% for OCT versus paraffin, and from 32 to 37% for MCH versus OCT. The major effect in innate absolute preference also came from the additive genetic contribution of the parental lines (Fig. 1, Table 1). Innate relative preference exhibited similar contributions of additive genetic effects and genetic specific interactions.

#### Resistance to infection

**Figure 2:**
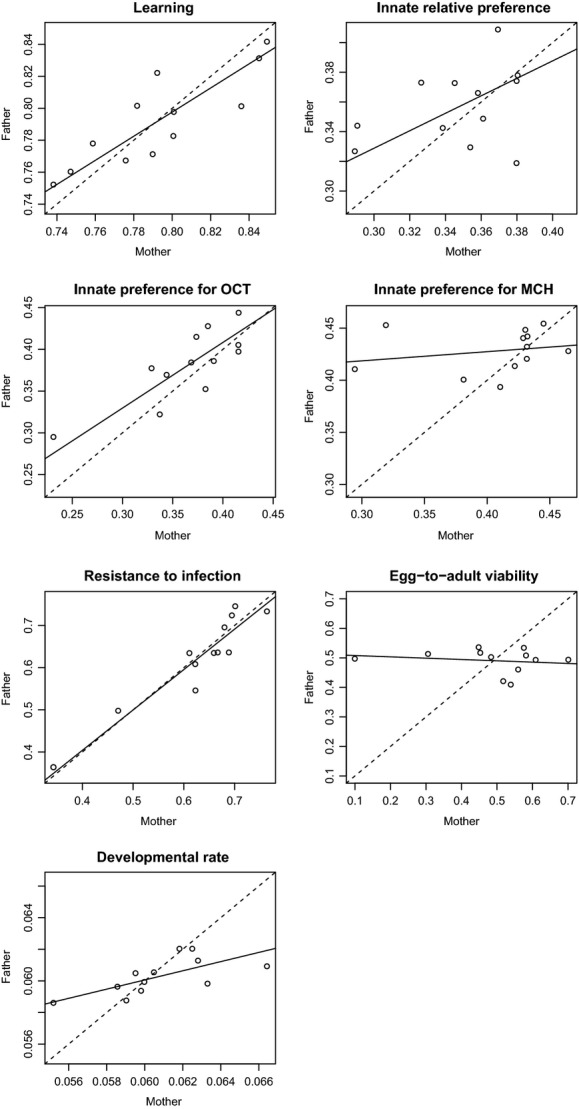
Mean phenotype of the inbred lines when used as sire lines against mean phenotype of the same lines when used as dams for each trait we measured, excluding inbred crosses. The solid line is the major axis regression, and the dashed line illustrates equal contributions to the phenotype of one line when used as sire and dam. When parental and maternal contributions are equivalent, observations are expected to lie on the diagonal line (slope of 1). A shallower relationship denotes a larger maternal contribution, whereas a steeper slope stands for a larger paternal contribution. The maternal contribution is larger in particular for larval viability and developmental rate on poor food. The maternal contribution seems to be large for innate preference for MCH, but this effect disappears when the analysis integrates other factors. From left to right and up to down: 1 – frequency of choosing the learned odor, 2 – frequency of choosing octanol over MCH, 3 – frequency of choosing octanol over pure paraffin, 4 – frequency of choosing MCH over pure paraffin, 5 – frequency of survival a bacterial infection, 6 – proportion of emergence, and 7 – developmental rate (1/days).

As for the above traits, survival after bacterial infection mostly depended on additive genetic effects. Genetic differences between lines were large, as survival frequencies ranged from 39 to 78%. Genetic specific interaction and reciprocal effects were weak. Similarly, parental effects were very small (Figs 1 and 2, Table 1). Variance due to differences between experimenters contributed strongly to the total variance, presumably because of differences in speed at administering infection by pricking.

#### Tolerance to malnutrition

Viability and developmental rate in low-food conditions varied significantly among lines; viability ranged from 25 to 57%, and development took between 15.8 and 17.8 days on average. Both traits feature a clear low-fitness outlier, but interestingly, the inbred line displaying a very low viability is not the same as the one with the slowest development. These lines are close to the average for the other traits.

Compared to other sources of variation, the contribution of the additive genetic variance was relatively modest in egg-to-adult viability and developmental rate assessed under poor food conditions. For these traits, the magnitude of parental effects was similar to additive effects (Table1, Fig. 1). Maternal line had a much stronger effect on mean offspring phenotype than the paternal line, in particular for egg-to-adult viability (Fig. 2); hence, the parental effects can be attributed mostly to maternal effects. Genetic specific and reciprocal effects, as well as experimenter effect, were weak and not statistically significant.

### Additive genetic correlations between traits

**Figure 3:**
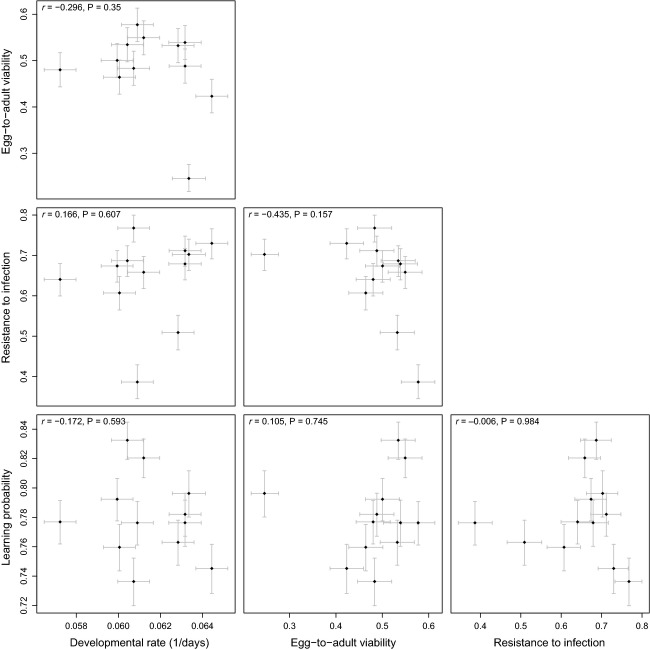
Additive genetic correlations between traits tested as correlations between additive effects (back-transformed to probabilities for binomial traits ± SE).

**Figure 4:**
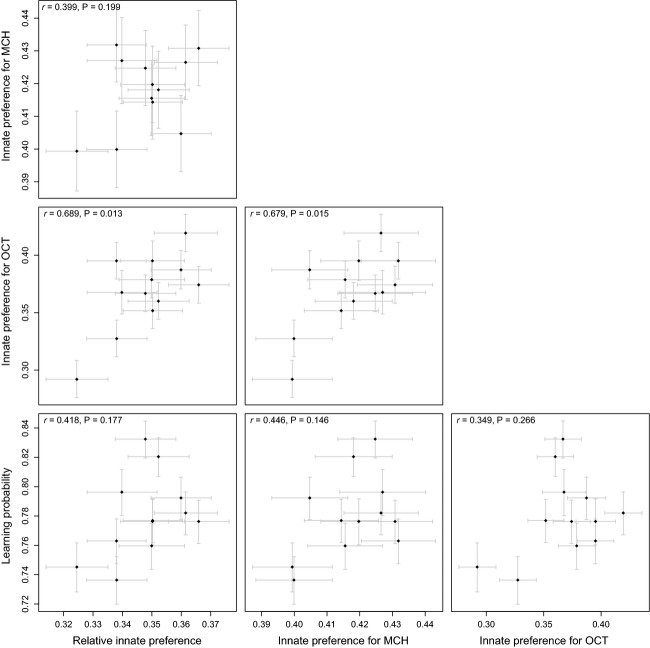
Additive genetic correlations between learning ability and innate preferences for odors (absolute innate preferences for OCT and MCH and relative innate preference between these two odors (proportions of flies choosing OCT were used as preference measures); back-transformed to probabilities ± SE).

None of the correlations between the additive effects of learning ability, resistance to infection, and the two measures of malnutrition tolerance (egg-to-adult viability and developmental rate on poor food) were statistically significant even before correction for multiple testing (Figs 3 and 4). In contrast, innate preferences are genetically correlated, suggesting that innate responses to different odors share some genetic basis.

### Inbreeding effect

Inbreeding was modeled through two complementary effects: the average effect of inbreeding (inbred parental lines vs. outbred) and the additional variance associated with inbreeding. Inbreeding affected almost all the traits we measured (Fig. 1). Learning ability of the outbred crosses was identical or better than in inbred parental lines (especially in the case of learning to avoid MCH). Parental lines seemed still to be able to innately avoid OCT or MCH, which are known to be aversive, but to a lesser extent than outbred flies. The relative innate preference was also affected by inbreeding effects; inbred flies seemed to be closer to a random choice (in this case, the average effect of inbreeding was greater than the range of natural variation in the population), which could indicate a decrease of their ability to smell and distinguish the odors. Flies from outbred crosses were more resistant to the bacterial infection. Egg-to-adult viability was also better in outbred crosses, and development was faster. The variance of inbreeding effect was non-null for egg-to-adult viability and, to a lesser extent, developmental rate, which suggests that for tolerance to malnutrition, the effect of inbreeding depends on the genotype.

## Discussion

### Origin of biological variation

We observed that the additive genetic variance 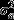 was higher than the genetic specific variance 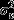 and the variance of reciprocal effects 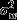 for each trait. The genetic specific variance 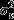 is a cross-specific variance, which represents the interaction between the contributions of the two parental lines. Due to the way inbreeding effects are represented in the model, 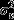 corresponds to interaction variances (dominance and epistasis) excluding inbreeding effects. High 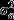/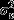 ratios indicate that the genotypic value of the cross progeny does not deviate much from the mean of the genotypic value of the parental lines for these traits. It also indicates a high broad-sense heritability for these traits.

For both traits which reflect malnutrition tolerance – egg-to-adult viability and developmental rate on poor food – the variance component attributable to parental effects, 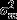, was similar in magnitude to the variance of genetic effects. This is consistent with previous studies, which found large maternal effects on larval development under malnutrition conditions (Vijendravarma et al. 2010; Vijendravarma and Kawecki 2013). This is not surprising when considering the uniparental (maternal) heredity of the egg cytoplasm, which is part of the environment for gene expression and nutrition in the early stages of development. Females from different inbred lines likely invest differently in their eggs in terms of nutrients, and/or provision the eggs differently with maternal-origin mRNAs and regulatory molecules such as microRNAs, leading to between-line variation in embryonic and larval development.

By contrast, 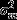, the variance due to parental effects, was virtually nil for all traits measured after development is completed, that is, learning performance, innate absolute, and relative preferences for odors and resistance to infection. Hence, phenotypes of the progeny were more under the control of the genetic contributions of their parents than under the control of maternal effects for these traits. This suggests that the role of genetic maternal effects fades at adult stage. It also dismisses a potential impact of Wolbachia infection status (unknown in our study) on the immune response to bacterial infection. Nevertheless, a recent study in *Drosophila* (Burns and Mery 2010) showed an effect of the mother age on the progeny learning performance in the same conditioning procedure, indicating that nongenetic maternal effects can have a significant effect on learning ability even at the adult stage.

### Additive genetic correlations between traits

None of the estimated genetic correlations between learning and life-history traits was significant. The absence of significant additive genetic correlation suggests the absence of systematic pleiotropic relationships between the two traits (Falconer and Mackay 1996). This result, however, does not formally prove that life-history traits and learning are under the control of nonoverlapping sets of genes. For instance, a pair of traits controlled by both synergistic and antagonistic pleiotropic genes may not display any genetic or phenotypic correlation.

A previous study (Nepoux et al. 2010) reported a significant positive correlation between learning performance and egg-to-adult viability on poor food across a set of *Drosophila* inbred lines which included the 12 lines used here. However, that result does not contradict the present findings: even though the genetic correlation between these two traits was not significant in the present work, the two correlation coefficient estimates are not significantly different from each other, as their 95 % confidence intervals largely overlap (present study: [−0.50; 0.64]; Nepoux et al. 2010: [0.22; 0.88]). Three inbred lines were lost between both experiments, and correlations were computed on 12 lines in the current study (instead of 15 in Nepoux et al. 2010), leading to some loss of power. With the current setting (12 data points), it is thus possible that a moderate correlation could have remained undetected (only correlations greater than *r* = 0.47 would have been detected with a probability >95%).

This lack of negative correlations between learning and fitness components stands in contrast to two selection experiments which did find such a trade-off (Mery and Kawecki 2003; Kolss and Kawecki 2008). Possibly, this difference results from differences in the architecture of genetic variation between base populations from which the flies were derived. However, it also fits the general trend for selection experiments being more likely to reveal trade-offs than estimates of genetic correlations obtained from resemblance between relatives (Fry 2003).

Evidence for evolutionary trade-offs between learning and immune defense is equivocal. At the phenotypic (plastic) level, several studies in bees found that infection or immune system activation impairs their learning ability (Mallon et al. 2003; Riddell and Mallon 2006), but the opposite effect has been found in *Drosophila* (Babin et al. 2014a). The absence of additive genetic correlation between learning ability and resistance to bacterial infection is also consistent with previously published results in *Drosophila* (Kolss et al. 2006) and in bumble bees (Alghamdi et al. 2009). Similarly, no genetic trade-off was detected between learning performance and tolerance to nutritional stress across inbred lines of *Drosophila* (Nepoux et al. 2010). Thus, it still remains open how often learning performance is genetically correlated with tolerance to nutritional and immune stress.

### Inbreeding depression

Inbreeding depression impairs many fitness-related traits (Wright 1977; Charlesworth and Charlesworth 1987; Crnokrak and Roff 1999), and a previous comparison between inbred lines and an outbred population has revealed moderate inbreeding depression in learning ability and tolerance to larval malnutrition in *Drosophila* (Nepoux et al. 2010). As the most deleterious alleles may have been purged during the generation of inbred lines, inbreeding depression could potentially be even higher than measured, at least for fitness-related traits. Purging of recessive deleterious alleles could also bias downwards the estimate of dominance variance for fitness traits.

In the present study inbreeding depression is manifested as a better performance of crosses between different inbred lines (i.e., heterosis), relative to offspring of mating within lines. This approach revealed inbreeding depression in all traits except learning to avoid octanol. The progeny of outbred crosses showed better learning performance on MCH as well as higher viability and faster development when raised on low-quality food. This is consistent with the presence of recessive deleterious alleles for this trait in our inbred lines.

Similarly, resistance to bacterial infection was also affected by inbreeding depression. This is consistent with known results on the effects of heterozygosity on resistance to infection (e.g., for MHC in vertebrates, reviewed in Wegner et al. 2004). This is also consistent with alleles that impair these aspects of performance being on average at least partially recessive.

### Maintenance of genetic variation

The results of the diallel crosses show that there is ample genetic variation for learning traits in natural populations. Furthermore, this genetic variance is mostly additive, which suggests that learning is evolvable and would respond to selection, provided a sufficient selection gradient. These findings are consistent with previous artificial selection experiments in *Drosophila melanogaster* (Mery and Kawecki 2002; Lofdahl et al. 1992) and in the honeybee *Apis mellifera* (Brandes, [Bibr b6],[Bibr b7]; Chandra et al. 2000). Understanding the maintenance of genetic variation in populations is still a challenging question in evolutionary biology (Barton and Turelli 1989; Barton and Keightley 2002; Johnson and Barton 2005) and probably involves several mechanisms, including mutation, genetic drift, and complex patterns of selection (Charlesworth and Hughes 2000). The popular alternatives to explain the presence of genetic variance in populations can be classified in three main categories: (1) balancing selection, (2) mutation-selection balance, and (3) antagonistic pleiotropy. Our results featuring large additive/dominance variance ratios clearly exclude balancing selection, which expects dominance to be a major component of genetic variation. Discarding the alternative explanations appears more speculative.

If the observed standing additive genetic variation results from a mutation-selection balance, the continuous loss of deleterious genetic variants needs to be compensated by new mutations. The link between natural selection and the amount of genetic variance is supported by the fact that traits measured after sexual maturity (resistance to infection and learning) tend to display more additive genetic variance than larval traits (developmental time and viability), which is theoretically predicted by some mutation-selection models (Charlesworth and Hughes 1996). However, maintaining such a large amount of additive variance requires unexpected low selection on fitness-related traits, and/or a large mutational variance. Although our data do not allow to reject the mutation-selection balance hypothesis, alternative or complementary hypotheses deserve serious consideration. In particular, a large amount of maladaptive genetic variation can be more realistically attributed to spatial heterogeneity in selection, if migration rates are high enough to introduce alleles from nearby populations with a different fitness optimum. Finally, one cannot exclude that the genetic variance in wild populations is actually small and that genetic-by-environment interactions could amplify or reveal cryptic genetic variation in laboratory conditions (Le Rouzic and Carlborg 2008).

Negative genetic correlations between fitness-related traits are thought to favor the maintenance of genetic variation and result in evolutionary trade-offs between traits (Rose and Charlesworth 1981; Rose 1982). However, the fact that we were unable to detect any genetic correlation between learning and various fitness-related traits does not support the trade-off hypothesis. If such correlations exist, they are necessarily weak, as our experimental design allows to detect almost systematically (>95%) any correlation higher than 0.47. Of course, the number of fitness traits were limited by logistic considerations, and it is possible that learning affects fitness negatively through untested fitness components (such as sexual selection). However, a strong, unidentified association between learning and fitness cannot explain the high amount of additive genetic variation we measured experimentally, as selection on fitness is necessarily strong.

A moderate amount of pleiotropy between learning and fitness components should slow down the evolution of the trait, but cannot constrain it totally. The presence of additive genetic variation and the absence of direct or indirect measurable selection to decrease learning are thus not compatible with the apparent stability of learning capacities in animals. This issue is far from being restricted to learning in insects; the lack of evolution in variable morphological traits is often referred to as the “paradox of stasis" (Hansen and Houle 2004; Estes and Arnold 2007). Here, we show that this paradox of stasis also affects behavioral traits, which could be a key information for a better understanding of the evolution of phenotypic plasticity.

## References

[b1] Alghamdi A, Raine NE, Rosato E, Mallon EB (2009). No evidence for an evolutionary trade-off between learning and immunity in a social insect. Biol. Lett.

[b2] Babin A, Kolly S, Schneider F, Dolivo V, Zini M, Kawecki TJ (2014a). Fruit flies learn to avoid odours associated with virulent infection. Biol. Lett.

[b3] Babin A, Kolly S, Kawecki TJ (2014b). Virulent bacterial infection improves aversive learning performance in *Drosophila melanogaster*. Brain Behav. Immun.

[b4] Barton N, Turelli M (1989). Evolutionary quantitative genetics: how little do we know?. Ann. Rev. Genet.

[b5] Barton NH, Keightley PD (2002). Understanding quantitative genetic variation. Nat. Rev. Genet.

[b6] Brandes C (1988). Estimation of heritability of learning behavior in honeybees (*Apis mellifera capensis*. Behav. Genet.

[b7] Brandes C (1991). Genetic differences in learning behavior in honeybees (*Apis mellifera capensis*. Behav. Genet.

[b8] Burger JM, Kolss M, Pont J, Kawecki TJ (2008). Learning ability and longevity: a symmetrical evolutionary trade-off in *Drosophila*. Evolution.

[b9] Burnham KP, Anderson DR (2002). Model selection and multi-model inference: a practical information-theoretic approach.

[b10] Burns JG, Mery F (2010). Transgenerational memory effect of ageing in *Drosophila*. J. Evol. Biol.

[b11] Chandra SB, Hosler JS, Smith BH (2000). Heritable variation for latent inhibition and its correlation with reversal learning in honeybees (*Apis mellifera*. J. Comp. Psychol.

[b12] Charlesworth B, Hughes KA (1996). Age-specific inbreeding depression and components of genetic variance in relation to the evolution of senescence. Proc. Natl Acad. Sci. USA.

[b13] Charlesworth B, Singh RamaS, Krimbas CostasB, Hughes KA (2000). The maintenance of genetic variation in life-history traits. Evolutionary genetics: from molecules to morphology, volume 1.

[b14] Charlesworth D, Charlesworth B (1987). Inbreeding depression and its evolutionary consequences. Ann. Rev. Ecol. Syst.

[b15] Cockerham CC, Weir BS (1977). Quadratic analyses of reciprocal crosses. Biometrics.

[b16] Crnokrak P, Roff DA (1999). Inbreeding depression in the wild. Heredity.

[b17] Danchin E, Blanchet S, Mery F, Wagner R (2010). Do invertebrates have culture?. Commun. Integr. Biol.

[b18] David JR, Clavel MF (1965). Intéraction entre le génotype et le milieu d'élevage, conséquences sur les caractéristiques de la drosophile. Bull. Biol. Fr. Belg.

[b19] Dubnau J, Tully T (1998). Gene discovery in *Drosophila*, new insights for learning and memory. Annu. Rev. Neurosci.

[b20] Dukas R (1998). Cognitive ecology.

[b22] Estes S, Arnold SJ (2007). Resolving the paradox of stasis: models with stabilizing selection explain evolutionary divergence on all timescales. Am. Nat.

[b23] Falconer DS, Mackay T (1996). Introduction to quantitative genetics.

[b24] Fry JD (2003). Detecting ecological trade-offs using selection experiments. Ecology.

[b25] Gegear RJ, Otterstatter MC, Thomson JD (2006). Bumble-bee foragers infected by a gut parasite have an impaired ability to utilize floral information. Proc. Biol. Sci.

[b26] Gibertini M, Newton C, Friedman H, Klein TW (1995). Spatial learning impairment in mice infected with *Legionella pneumophila* or administered exogenous interleukin-1-beta. Brain Behav. Immun.

[b27] Gimelfarb A (1989). Genotypic variation for a quantitative character maintained under stabilizing selection without mutations, epistasis. Genetics.

[b28] Greenberg AJ, Hackett SR, Harshman LG, Clark AG (2010). A hierarchical bayesian model for a novel sparse partial diallel crossing design. Genetics.

[b29] Griffing B (1956). Concept of general and specific combining ability in relation to diallel crossing systems. Aust. J. Biol. Sci.

[b30] Hansen TF, Pigliucci M, Preston K, Houle D (2004). Phenotypic integration: studying the ecology and evolution of complex phenotypes. Evolvability, stabilizing selection and the problem of stasis.

[b31] Jaumann S, Scudelari R, Naug D (2013). Energetic cost of learning and memory can cause cognitive impairment in honeybees. Biol. Lett.

[b32] Johnson T, Barton N (2005). Theoretical models of selection and mutation on quantitative traits. Philos. Trans. R. Soc. Lond. B Biol. Sci.

[b33] Kavaliers M, Colwell DD, Galea LAM (1995). Parasitic infection impairs spatial learning in mice. Anim. Behav.

[b34] Kawecki TJ (2010). Evolutionary ecology of learning: insights from fruit flies. Popul. Ecol.

[b35] Kawecki TJ, Mery F (2006). Genetically idiosyncratic responses of *Drosophila melanogaster* populations to selection for improved learning ability. J. Evol. Biol.

[b36] Kolss M, Kawecki TJ (2008). Reduced learning ability as a consequence of evolutionary adaptation to nutritional stress in *Drosophila melanogaster*. Ecol. Entomol.

[b37] Kolss M, Kraaijeveld AR, Mery F, Kawecki TJ (2006). No trade-off between learning ability and parasitoid resistance in *Drosophila melanogaster*. J. Evol. Biol.

[b41] Lenarcic AB, Svenson KL, Churchill GA, Valdar W (2012). A general Bayesian approach to analyzing diallel crosses of inbred strains. Genetics.

[b42] Lemaitre B, Hoffmann J (2007). The host defense of *Drosophila melanogaster*. Ann. Rev. Immunol.

[b43] Le Rouzic A, Carlborg Ö (2008). Evolutionary potential of hidden genetic variation. Trends Ecol. Evol.

[b44] Lofdahl KL, Holliday M, Hirsch J (1992). Selection for conditionability in *Drosophila melanogaster*. J. Comp. Psychol.

[b45] Lynch M, Walsh B (1998). Genetics and analysis of quantitative traits.

[b47] Mallon EB, Brockmann A, Schmid-Hempel P (2003). Immune response inhibits associative learning in insects. Proc. Biol. Sci.

[b48] Margulies C, Tully T, Dubnau J (2005). Deconstructing memory in *Drosophila*. Curr Biol.

[b49] Mery F, Kawecki TJ (2002). Experimental evolution of learning ability in fruit flies. Proc. Natl Acad. Sci. USA.

[b50] Mery F, Kawecki TJ (2003). A fitness cost of learning ability in *Drosophila melanogaster*. Proc. Biol. Sci.

[b51] Mery F, Kawecki TJ (2005). A cost of long-term memory in *Drosophila*. Science.

[b52] Mery F, Pont J, Preat T, Kawecki TJ (2007). Experimental evolution of olfactory memory in *Drosophila melanogaster*. Physiol. Biochem. Zool.

[b54] Nepoux V, Haag CR, Kawecki TJ (2010). Effects of inbreeding on aversive learning in *Drosophila*. J. Evol. Biol.

[b55] Papaj DR, Prokopy RJ (1989). Ecological and evolutionary aspects of learning in phytophagous insects. Ann. Rev. Entomol.

[b56] R Core Team (2014). R: a language and environment for statistical computing.

[b57] Rescorla RA (1988). Behavioral studies of pavlovian conditioning. Annu. Rev. Neurosci.

[b58] Riddell CE, Mallon EB (2006). Insect psychoneuroimmunology: immune response reduces learning in protein starved bumblebees (*Bombus terrestris*. Brain Behav. Immun.

[b59] Roff DA, Emerson K (2006). Epistasis and dominance: evidence for differential effects in life-history versus morphological traits. Evolution.

[b60] Rönnegård L, Shen X, Alam M (2010). hglm: a package for fitting hierarchical generalized linear models. R J.

[b61] Rose MR (1982). Antagonistic pleiotropy, dominance, and genetic variation. Heredity.

[b62] Rose MR, Charlesworth B (1981). Genetics of life history in *Drosophila melanogaster*. I. Sib analysis of adult females. Genetics.

[b63] Sheather SJ, Jones MC (1991). A reliable data-based bandwidth selection method for kernel density estimation. J. Roy. Stat. Soc. B.

[b64] Shettleworth SJ (1998). Cognition, evolution and behaviour.

[b65] Sparkman NL, Kohman RA, Garcia AK, Boehm GW (2005). Peripheral lipopolysaccharide administration impairs two-way active avoidance conditioning in c57bl/6j mice. Physiol. Behav.

[b66] Sprague GF, Tatum LA (1942). General vs. specific combining ability in single crosses of corn. J. Am. Soc. Agron.

[b67] Stearns SC (1989). Trade-offs in life-history evolution. Func. Ecol.

[b68] Tully T, Preat T, Boynton SC, Del Vecchio M (1994). Genetic dissection of consolidated memory in *Drosophila*. Cell.

[b69] Vaida F, Blanchard S (2005). Conditional Akaike information for mixed-effects models. Biometrika.

[b70] Vijendravarma RK, Kawecki TJ (2013). Epistasis and maternal effects in experimental adaptation to chronic nutritional stress in *Drosophila*. J. Evol. Biol.

[b71] Vijendravarma RK, Narasimha S, Kawecki TJ (2010). Effects of parental larval diet on egg size and offspring traits in *Drosophila*. Biol. Lett.

[b72] Vodovar N, Vinals M, Liehl P, Basset A, Degrouard J, Spellman P, Boccard F, Lemaitre B (2005). *Drosophila* host defense after oral infection by an entomopathogenic pseudomonas species. Proc. Natl Acad. Sci. USA.

[b73] Wegner KM, Kalbe M, Schaschl H, Reusch TBH (2004). Parasites and individual major histocompatibility complex diversity–an optimal choice. Microbes Infect.

[b74] Wricke G, Weber WE (1986). Quantitative genetics and selection in plant breeding.

[b75] Wright S (1977). Evolution and the genetics of populations, Vol 3, experimental results, and evolutionary deductions.

[b76] Zemach A, McDaniel IE, Silva P, Zilberman D (2010). Genome-wide evolutionary analysis of eukaryotic DNA methylation. Science.

